# Intragenic meiotic recombination in *Schizosaccharomyces pombe* is sensitive to environmental temperature changes

**DOI:** 10.1007/s10577-020-09632-3

**Published:** 2020-04-17

**Authors:** Simon D. Brown, Charlotte Audoynaud, Alexander Lorenz

**Affiliations:** 1 The Institute of Medical Sciences (IMS), University of Aberdeen, Foresterhill, Aberdeen, AB25 2ZD UK; 2 Present Address: MRC Institute of Genetics & Molecular Medicine, University of Edinburgh, Edinburgh, EH4 2XU UK; 3 Present Address: Institut Curie, PSL Research University, UMR3348-CNRS, 91405 Orsay, France

**Keywords:** meiosis, meiotic recombination, environmental temperature, *Schizosaccharomyces pombe*

## Abstract

Changes in environmental temperature influence cellular processes and their dynamics, and thus affect the life cycle of organisms that are unable to control their cell/body temperature. Meiotic recombination is the cellular process essential for producing healthy haploid gametes by providing physical links (chiasmata) between homologous chromosomes to guide their accurate segregation. Additionally, meiotic recombination—initiated by programmed DNA double-strand breaks (DSBs)—can generate genetic diversity and, therefore, is a driving force of evolution. Environmental temperature influencing meiotic recombination outcome thus may be a crucial determinant of reproductive success and genetic diversity. Indeed, meiotic recombination frequency in fungi, plants and invertebrates changes with temperature. In most organisms, these temperature-induced changes in meiotic recombination seem to be mediated through the meiosis-specific chromosome axis organization, the synaptonemal complex in particular. The fission yeast *Schizosaccharomyces pombe* does not possess a synaptonemal complex. Thus, we tested how environmental temperature modulates meiotic recombination frequency in the absence of a fully-fledged synaptonemal complex. We show that intragenic recombination (gene conversion) positively correlates with temperature within a certain range, especially at meiotic recombination hotspots. In contrast, crossover recombination, which manifests itself as chiasmata, is less affected. Based on our observations, we suggest that, in addition to changes in DSB frequency, DSB processing could be another temperature-sensitive step causing temperature-induced recombination rate alterations.

**Electronic supplementary material:**

The online version of this article (10.1007/s10577-020-09632-3) contains supplementary material, which is available to authorized users.

## Introduction

Single-celled organisms, such as yeasts, and other organisms unable to control their internal temperature are at the mercy of the environmental temperature, which can fluctuate considerably between seasons and during day-night cycles. *Schizosaccharomyces pombe* and *Saccharomyces cerevisiae* are globally distributed and very distantly related species with a rather poorly understood ecology (Liti 2015; Jeffares 2018), but it is likely that they are exposed to changing temperatures in their respective niches. Changes in temperature can modulate meiotic recombination outcome in a range of organisms, including fungi (Plough 1917; Lu 1974; Rose and Baillie 1979; Börner et al. 2004; Pryce et al. 2005; Higgins et al. 2012; Phillips et al. 2015; Zhang et al. 2017; Lloyd et al. 2018; Modliszewski et al. 2018). Accordingly, environmental temperature has been suggested to be a major driver in the evolutionary adaptation of the meiotic machinery (Bomblies et al. 2015).

The main function of meiotic recombination is to ensure correct chromosome segregation, as it establishes physical connections between the homologous chromosomes (homologues). This is achieved through the repair of programmed DNA double-strand breaks (DSBs) from the homologue rather than the sister chromatid, and the processing of recombination intermediates between the homologues as crossovers (COs) (de Massy 2013; Hunter 2015). The transesterase Spo11 makes the DSBs, preferentially in particular regions called hotspots (de Massy 2013; Tock and Henderson 2018). Subsequently, the DSB ends are resected to enable homologous recombination, which ultimately leads to COs and non-crossovers (NCOs) depending on the repair pathway (de Massy 2013; Gray and Cohen 2016).

Meiotic recombination is influenced by the chromatin environment and in most organisms by the formation of a meiosis-specific axis along the chromosomes. These axes are then connected by filamentous proteins to build the synaptonemal complex (Hunter 2015; Gray and Cohen 2016). Proteins forming the chromosome axis and/or the synaptonemal complex are important to enable meiotic recombination or at least to maintain it at wild-type levels (de Massy 2013; Hunter 2015; Gray and Cohen 2016). A large part of the effect on meiotic recombination frequency or event placement exerted by temperature changes seems to be mediated by the chromosome axis and the synaptonemal complex in many organisms (Morgan et al. 2017). Fission yeast lacks a canonical synaptonemal complex (Olson et al. 1978; Bähler et al. 1993; Molnar et al. 2003). It only forms the so-called linear elements, which are meiotic chromosome axes evolutionarily related to the lateral elements of the synaptonemal complex (Lorenz et al. 2004; Loidl 2006, 2016). *Sz. pombe* is, thus, an ideal system to test the response of recombination to temperature changes in the absence of a fully-fledged synaptonemal complex.

Here, we employed a series of genetic and cytological assays to test whether fission yeast meiosis and meiotic recombination are susceptible to temperature changes. We determined the full ‘fertile range’ (Bomblies et al. 2015) of the *Sz. pombe* laboratory strain, and measured meiotic intra- and intergenic recombination frequencies at and around *ade6* using a genetic assay (Lorenz et al. 2010). We find that intergenic recombination around *ade6* is not strongly affected over the fertile range in *Sz. pombe*, but that gene conversion, especially at hotspots engendered by point mutants in *ade6*, is cold-sensitive. Further experimentation indicates that in addition to changes in the formation of DSBs (Hyppa et al. 2014), DSB processing could be another source for a reduced gene conversion frequency particularly at temperatures below 25 °C.

## Material and methods

### Yeast strains and culture conditions

Cells were cultured on yeast extract (YE), and on yeast nitrogen base glutamate (YNG) agar plates containing the required supplements (concentration 250 mg/l on YE, 75 mg/l on YNG). Crosses were performed on malt extract (ME) agar containing supplements at a final concentration of 50 mg/l (Sabatinos and Forsburg 2010).

All *Schizosaccharomyces pombe* strains used for this study were either published previously, or have been generated from existing strains by crossing (see Table S1). Different *ade6* alleles (Table S2) were introduced by crossing the respective mutant *ade6* strain with *ade6*^+^ strains carrying the *ura4*^+^ and *his3*^+^ artificially introduced markers (aim) (UoA95, UoA96, UoA97, UoA98) (Osman et al. 2003). The point mutations in the *ade6* alleles were verified by Sanger DNA sequencing (Source BioScience, Nottingham, UK) (Table S2).

Epitope tagging of *hop1*^+^ with a C-terminal *13myc-kanMX6* tag has been described in detail (Brown et al. 2018).

### Genetic and cytological assays

Determination of spore viability by random spore analysis and the meiotic recombination assay were performed as previously described (Osman et al. 2003; Sabatinos and Forsburg 2010).

Meiotic time-courses and preparation of chromatin spreads were in essence performed as published (Loidl and Lorenz 2009), except for the use of 100 mg/ml Lallzyme MMX (Lallemand Inc., Montréal, Canada) as the cell-wall digesting enzyme (Flor-Parra et al. 2014). Immunostaining was performed according to an established protocol (Loidl and Lorenz 2009) using polyclonal rabbit *α*-myc (ab9106; Abcam PLC, Cambridge, UK) at a 1:500 dilution and monoclonal rat *α*-GFP [3H9] (ChromoTek GmbH, Planegg-Martinsried, Germany) at a 1:100 dilution as primary antibodies. Antibody-bound protein was visualized using donkey *α*-rabbit IgG AlexaFluor-555 (ab150062; Abcam) and donkey *α*-rat IgG AlexaFluor-488 (ab150153; Abcam), both at a 1:500 dilution. DNA was stained by Hoechst 33342 (Molecular Probes, Eugene, OR, USA) at a final concentration of 1 μg/ml.

Black-and-white images were taken with a Zeiss AxioCam MRm CCD camera (controlled by AxioVision 40 software v4.8.2.0) mounted on a Zeiss Axio Imager.M2 (Carl Zeiss AG, Oberkochen, Germany) epifluorescence microscope equipped with the appropriate filter sets to detect red, green and blue fluorescence. Individual images were acquired for each channel to detect Hop1-13myc, Rec7-GFP, Rad11-GFP and Hoechst 33342. Images were pseudo-coloured and overlayed using Adobe Photoshop CC (Adobe Systems Inc., San José, CA, USA). Immunodetected Rec7-GFP and Rad11-GFP foci were counted on images of meiotic prophase I nuclei at the thread and network stages identified by the presence of immunostained Hop1-13myc linear elements (Lorenz et al. 2006) within the Hoechst 33342-positive area using the ‘count’ function in Adobe Photoshop CC.

### Data presentation and statistics

Raw data is available on figshare (10.6084/m9.figshare.11192861). Line graphs and bar charts were produced using Microsoft Excel 2016 (version 16.0.4638.1000, 32-bit), and scatter plots were generated in GraphPad Prism 5 for Windows (version 5.04). Box-and-whisker plots were created in R (version i386, 3.0.1) (http://www.r-project.org/) (Lorenz et al. 2014). R was also used to compute Kruskal-Wallis test and Tukey’s Honest Significant Differences employing the kruskal.test() and TukeyHSD() functions, respectively. Mann-Whitney U tests were performed as previously described (Lorenz et al. 2014).

## Results

### The fertile range of fission yeast lies between 11 and 33 °C

**Fig. 1:**
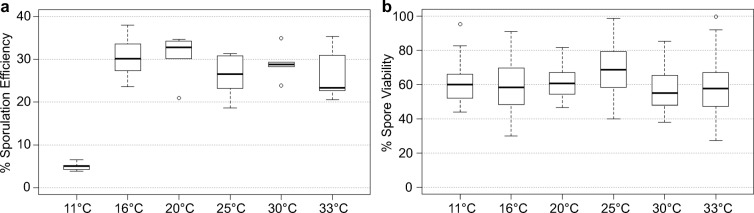
The fertile range of *Schizosaccharomyces pombe*. The upper, middle and lower lines of the box represent the third, second and first quartile, respectively (second quartile = median). The ‘whiskers’ represent the minimum and maximum of the range, unless they differ more than 1.5-times the interquartile distance from the median, then the 1.5-times interquartile distance around the median is indicated by the ‘whiskers’, and outliers are shown as open circles. **a** Sporulation efficiency in % determined in crosses of ALP714 × ALP688 at 11 °C after 14d (*n* = 7), at 16 °C after 7d (*n* = 6), at 20 °C after 5d (*n* = 5), at 25 °C after 3d (n = 6), at 30 °C after 2d (*n* = 6) and at 33 °C after 2d (*n* = 6). **b** Cumulative spore viability in % encompassing all data in Fig. 3 at 11 °C after 14d (*n* = 11), at 16 °C after 7d (*n* = 64), at 20 °C after 5d (*n* = 46), at 25 °C after 3d (*n* = 75), at 30 °C after 2d (*n* = 48) and at 33 °C after 2d (*n* = 59). For details of data see Tables S3 for (a) and S4 for (b)

Bomblies and co-workers recently noted that in order to understand temperature effects on meiotic recombination, it is important to know the ‘fertile range’ of a species; otherwise, the results will be skewed by including temperatures outside or omitting temperatures within the fertile range (Bomblies et al. 2015; Lloyd et al. 2018). We set up matings of prototrophic fission yeast strains (ALP714 × ALP688) in a temperature range between + 4 and + 35 °C on sporulation media. Matings were checked regularly whether asci containing spores were observed within 30 days. No asci were observed at + 4 and at + 35 °C after 1 month of incubation, putting the fertile range of *Sz. pombe* somewhere between these two temperatures. Indeed, mating at 11 °C resulted in the formation of asci containing viable spores within 14 days, at 16 °C within 7 days, at 20 °C within 5 days, at 25 and 30 °C within 3 days and at 33 °C within 2 days (Fig. 1).

Sporulation efficiency (i.e. the percentage of asci containing spores among the total population of cells) was ~ 25% at all temperatures, except at 11 °C when it was only ~ 5% (Fig. 1a, Table S3).

**Fig. 2:**
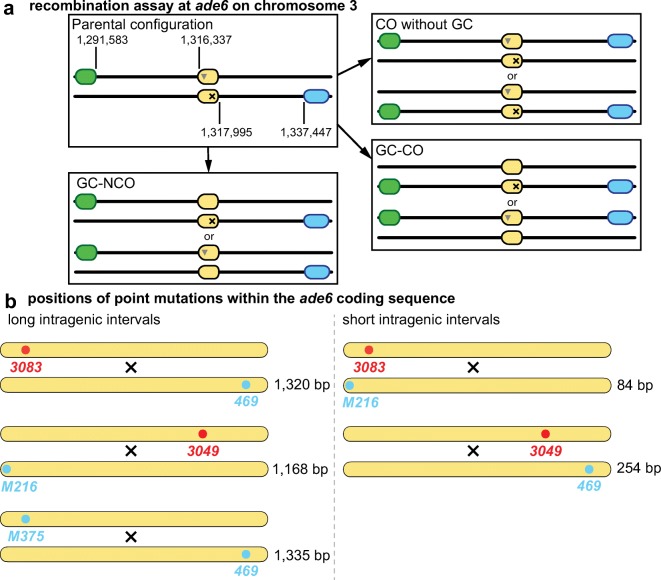
Meiotic recombination assay composed of different *ade6* alleles flanked by artificially introduced markers *ura4*^+^-*aim2* & *his3*^+^-*aim*. **a** Schematic showing the meiotic recombination assay at *ade6* (yellow) and its common outcomes. Ade^+^ recombinants arise via gene conversion (GC) associated with a crossover (GC-CO) or a non-crossover (GC-NCO). The positions of *ade6* and the artificially introduced markers *ura4*^+^-*aim2* (green) and *his3*^+^-*aim* (light blue) on chromosome 3 are indicated [in bps]. Positions of point mutations are shown as ▼ and ×. **b** Schematic of the *ade6* coding sequence indicating the point mutations and their positions (approximately to scale) used in the recombination assays, hotspots are indicated in red and non-hotspots in light blue. The distance between the sequence polymorphisms across the homologues is indicated in relation to the given hotspot of each cross [in bp]

**Fig. 3:**
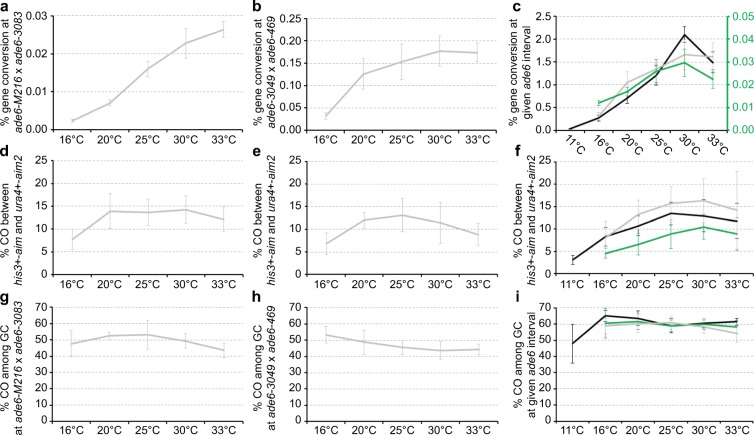
Environmental temperature alters recombination frequency at *ade6*. Frequency of **a**–**c** gene conversion in wild type at the indicated intragenic *ade6* interval, **d**–**f** crossover (CO) between *his3*^+^-*aim* and *ura4*^+^-*aim2* and **g**–**i** CO between *his3*^+^-*aim* and *ura4*^+^-*aim2* among gene conversion (GC) events at *ade6* from crosses performed at different temperatures. **(a**, **d**, **g)** UoA110 × UoA100: 16 °C (*n* = 15), 20 °C (*n* = 10), 25 °C (*n* = 12), 30 °C (*n* = 12), 33 °C (*n* = 12). **(b**, **e**, **h)** UoA120 × ALP731: 16 °C (*n* = 8), 20 °C (*n* = 8), 25 °C (*n* = 31), 30 °C (*n* = 8), 33 °C (*n* = 8). **(c**, **f**, **i)** UoA99 × UoA123 (*ade6-M216* × *ade6-3049*, grey line): 16 °C (*n* = 18), 20 °C (*n* = 12), 25 °C (*n* = 12), 30 °C (*n* = 17), 33 °C (*n* = 17); ALP733 × ALP731 (*ade6-3083* × *ade6-469*, black line): 11 °C (*n* = 11), 16 °C (*n* = 12), 20 °C (*n* = 14), 25 °C (*n* = 20), 30 °C (*n* = 12), 33 °C (*n* = 11); ALP1541 × ALP731 (*ade6-M375* × *ade6-469*, green line) 16 °C (*n* = 12), 20 °C (*n* = 12), 25 °C (*n* = 16), 30 °C (*n* = 12), 33 °C (*n* = 11). In (c) values for the green line are to be read from green secondary y-axis. *n* indicates the number of independent crosses. For details of data see Table S4

We also monitored spore viability by random spore analysis (Fig. 1b) during the following meiotic recombination assays (Figs. 2 and 3) performed at 11, 16, 20, 25, 30 and 33 °C (within the fertile range). At all temperatures tested, spore viability was ~ 60% (Fig. 1b), indicating that even at 11 °C when sporulation was rather inefficient (Fig. 1a), the viability of the spores that did develop was normal.

### The meiotic recombination assay

Our meiotic recombination assay (Osman et al. 2003; Lorenz et al. 2014; Brown et al. 2019) consists of intragenic markers (point mutations in the *ade6* gene) and the intergenic markers *ura4*^*+*^*-aim2* and *his3*^*+*^*-aim*, which are introduced to flank the *ade6* gene (Fig. 2). With this assay, various recombination outcomes can be monitored simultaneously: (I) intragenic recombination (gene conversion) events producing Ade^+^ recombinants, (II) COs between the flanking markers and (III) the ratio of COs vs. NCOs among *ade6*^+^ gene conversions (Fig. 2a). Gene conversion and overall CO rates are determined by DSB frequencies and the choice of repair template (homologous chromosome vs. sister chromatid). After strand exchange between homologous chromosomes and recombination intermediate processing, an *ade6*^+^ gene conversion event (non-reciprocal exchange of hereditary information) can be produced, which may be accompanied by either a CO or a NCO.

### Meiotic intragenic recombination levels vary greatly within the fertile range

To assess whether temperature alters meiotic recombination outcome, assays were performed at temperatures within the fertile range (Figs 2 and 3). We tested five different combinations of *ade6* heteroalleles: two large intragenic intervals containing an *M26*-type hotspot allele (*ade6-3083* × *ade6-469*, 1320 bp and *ade6-M216* × *ade6-3049*, 1168 bp), one large intragenic interval containing non-hotspot alleles only (*ade6-M375* × *ade6-469*, 1335 bp) and two small intragenic intervals containing an *M26*-type hotspot allele (*ade6-M216* × *ade6-3083*, 85 bp and *ade6-3049* × *ade6-469*, 254 bp) (Fig. 2b) (Lorenz et al. 2010, 2012; Brown et al. 2019). The *ade6-M26* hotspot and its variants, *ade6-3049*, *ade6-3083* and *ade6-3074*, are a well-studied type of meiotic recombination hotspot (Steiner and Smith 2005; Wahls and Davidson 2012). They contain the DNA sequence 5’-ATGACGT-3’ which generates a binding site for the transcription factor Atf1-Pcr1 (reviewed in Wahls and Davidson 2012).

The frequencies of gene conversion at both *M26*-type hotspots and the *ade6-M375* non-hotspot are considerably lower at colder temperatures (11 °C, 16 °C and 20 °C), and tend to plateau between 25 and 33 °C (Fig. 3a–c). One of the large intervals (*ade6–3083* × *ade6-469*) displayed a distinct peak at 30 °C (*p* = 2.67 × 10^−11^ 25 °C vs. 30 °C, *p* = 2.6 × 10^−5^ 30 °C vs. 33 °C; two-tailed Mann-Whitney U test) (Fig.3c). Intriguingly, the fold-change in intragenic recombination frequency between 16 °C (lowest temperature tested in all intervals) and the temperature producing the highest intragenic recombination frequency is substantially lower in the cross with the *ade6-M375* non-hotspot (2.7-fold) than in the crosses containing a hotspot allele (5- to 11-fold) (Table S4). This also holds true if gene conversion frequency is compared between 16 and 25 °C (the mating temperature generally used for this type of experiment): 2.4-fold change in *ade6-M375* × *ade6-469* vs. a 4.3- to 6.6-fold change in the crosses containing a hotspot allele (Table S4). The very short *ade6-M216* × *ade6-3083* intragenic interval (85 bp) shows a stronger fold-change over temperature (6.6-fold at 16 °C vs. 25 °C), than the longer intervals containing a hotspot allele (254–1320 bp; 4.3- to 4.8-fold at 16 °C vs. 25 °C) (Table S4). This suggests, (I) that, as a general trend, lower temperatures reduce the frequency of intragenic recombination regardless of physical distance between *ade6* mutations, (II) that gene conversion at the *ade6-M375* non-hotspots is less sensitive to temperature changes than at hotspots and (III) that intragenic recombination at very short intervals within *ade6* is singularly susceptible to temperature changes.

### Meiotic CO frequency varies moderately within the fertile range

Given that major changes in gene conversion, levels are observed across temperatures, we were surprised to find that both the overall CO levels and the CO frequencies among intragenic Ade + events were less sensitive to temperature changes. The frequency of COs between *ura4 + -aim2* and *his3 + -aim* are not substantially altered as temperature changes (Fig. 3d–f). In all intervals tested, CO frequency in the total population is only significantly lower at the temperatures of 11 and 16 °C, but then plateaus at 20 °C and higher (Fig. 3d–f, Table S5; Tukey’s Honest Significant Differences). CO frequency among *ade6*^+^ gene conversion events was even more stable with temperature changes (Fig. 3g–i). The non-hotspot only cross *ade6-M375* × *ade6-469* was completely unfazed by temperature changes (*p* = 0.314, Kruskal-Wallis test; Table S5). The crosses at cold temperatures (11, 16 and 20 °C) in all the other intervals displayed a moderate tendency to higher CO percentages than crosses at 30 °C or 33 °C (Fig. 3g–i, Table S5; Tukey’s Honest Significant Differences). The latter observation could be explained by a mechanism like CO homeostasis (Martini et al. 2006; Kan et al. 2011).

### Meiotic DSB levels do not appear to change with temperature

Following the observation that temperature modulates meiotic recombination outcome, we next sought to pinpoint which specific steps during meiotic recombination are sensitive to temperature changes. Because *Sz. pombe* does not have a canonical synaptonemal complex (Loidl 2006), other meiotic features must be responsible for the changes in recombination outcome in response to temperature (Morgan et al. 2017). First, we assessed whether DSB formation is perturbed using the cytological markers Rec7-GFP and Rad11-GFP. Rec7 (Rec114 in *S. cerevisiae*), one of the co-factors essential for Spo11 recruitment and function (Molnar et al. 2001; Miyoshi et al. 2013), can be detected on meiotic chromatin and is considered a marker for DSB initiation sites (Lorenz et al. 2006). As part of RPA (replication protein A), Rad11 becomes associated with the single-stranded DNA exposed by strand resection following removal of Spo11, and is, thus, a marker for DSB formation (Parker et al. 1997). Thus, Rec7- and Rad11-focus numbers allow the indirect assessment of meiotic DSB levels. For Rec7- and Rad11-focus counts, linear elements outlined by myc-tagged Hop1 were used to identify meiotic prophase I nuclei in chromatin spreads from meiotic time-courses (Lorenz et al. 2004, 2006; Loidl and Lorenz 2009; Brown et al. 2018). To avoid potential issues of tags affecting protein functionality, we employed all tagged genes heterozygously (Lorenz et al. 2006; Brown et al. 2018); both strains (UoA825 and UoA826) behaved normally in terms of meiotic progression and sporulation efficiency during meiotic timecourses (data not shown). We chose to perform these experiments at the extreme temperatures of the fertile range (16 and 33 °C), which result in significantly different recombination frequencies at a high sporulation efficiency (Figs. 1 and 3).

**Fig. 4:**
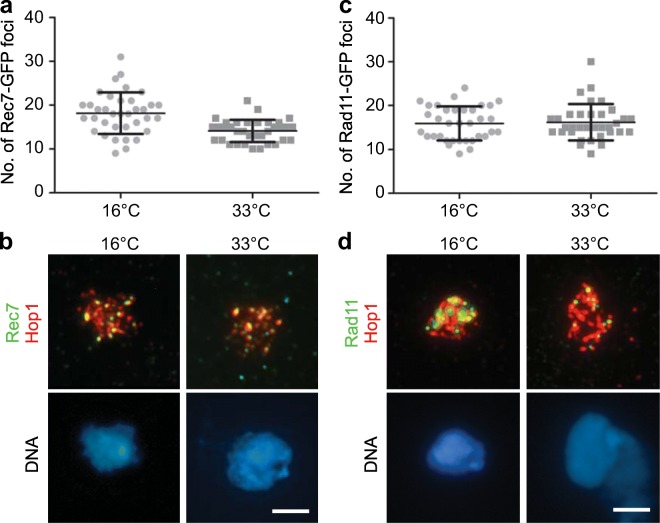
DSB formation does not seem to be affected by temperature. Focus counts of immune-detected Rec7-GFP and Rad11-GFP on Hop1-positive nuclear spreads from meiotic, azygotic timecourses at timepoints with maximum horsetail (meiotic prophase I) nucleus frequency; black horizontal lines indicate mean values, error bars represent standard deviation; for details of data see Table S6. **a** Number of Rec7-GFP foci per nucleus at 16 °C (25 h timepoint, *n* = 36) and 33 °C (5 h timepoint, *n* = 35) from meiotic timecourses of UoA825. **b** Examples of chromatin spreads evaluated in (a), Rec7-GFP in green, Hop1-13myc in red and DNA stained by Hoechst 33342 in blue; scale bar represents 5 μm. **c** Number of Rad11-GFP foci per nucleus at 16 °C (25 h timepoint, *n* = 35) and 33 °C (5 h timepoint, *n* = 35) from meiotic timecourses of UoA826. **d** Examples of chromatin spreads evaluated in (c), Rad11-GFP in green, Hop1-13myc in red and DNA stained by Hoechst 33342 in blue; scale bar represents 5 μm

Based on previous observations that recombination markers are most abundant in the thread and network stage of linear element formation (Lorenz et al. 2006), we selectively counted foci at these stages. On average ~ 16 foci per nucleus of Rec7-GFP and Rad11-GFP were observed at 16 and 33 °C (Fig. 4). The Rec7-GFP focus count was actually somewhat higher at the lower temperature (18.2 at 16 °C vs. 14.1 at 33 °C, *p* = 0.0017, two-tailed Mann-Whitney U test), whereas the Rad11-GFP focus numbers were indiscernible between 16 °C (15.9 foci/nucleus) and 33 °C (16.2 foci/nucleus) (Fig. 4, Table S6; *p* = 0.794, two-tailed Mann-Whitney U test).

These experiments suggest that overall DSB formation is possibly unaltered between 16 and 33 °C, because any of the subtle changes observed are unlikely to explain the lowered recombination frequencies at cold temperatures.

### Processing of DSBs is potentially altered by temperature

**Fig. 5:**
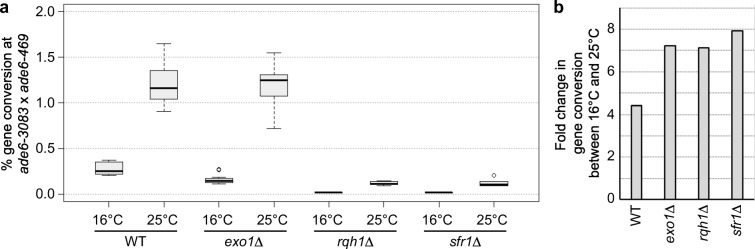
Cold temperature causes stronger reductions in gene conversion frequency in *exo1*, *rqh1* or *sfr1* deletions than in wild type. **a** Frequency of gene conversion at *ade6-3083* × *ade6-469* at 16 °C and 25 °C in wild type (WT), *exo1*Δ, *rqh1*Δ and *sfr1*Δ. ALP733 × ALP731 (WT; 16 °C *n* = 12, 25 °C *n* = 20), MCW4269 × MCW4268 (*exo1*Δ; 16 °C *n* = 11, 25 °C *n* = 11), ALP781 × ALP780 (*rqh1*Δ; 16 °C *n* = 12, 25 °C *n* = 10), ALP800 × ALP782 (*sfr1*Δ; 16 °C *n* = 11, 25 °C *n* = 10). *n* indicates the number of independent crosses. The upper, middle and lower lines of the box represent the third, second and first quartile, respectively (second quartile = median). The ‘whiskers’ represent the minimum and maximum of the range, unless they differ more than 1.5-times the interquartile distance from the median, then the 1.5-times interquartile distance around the median is indicated by the ‘whiskers’, and outliers are shown as open circles. **b** Fold change of data in (a). For details of data see Table S4

Rqh1 and Exo1 function in long-range strand resection in mitosis and meiosis in fission yeast (Langerak et al. 2011; Osman et al. 2016). Sfr1 forms a complex with Swi5 to support strand exchange, thereby promoting meiotic recombination (Ellermeier et al. 2004; Haruta et al. 2006; Lorenz et al. 2012). Less efficient DNA resection and/or reduced strand exchange with the homologous chromosomes could potentially explain why recombination levels are reduced at colder temperatures. The expectation would be that mutants defective in strand resection or strand exchange would be more sensitive to temperature changes than wild type (i.e. a synergistic effect of mutational and environmental weakening of these processes). Therefore, meiotic recombination outcome in *ade6-3083* × *ade6-469* crosses of *rqh1*Δ, *exo1*Δ and *sfr1*Δ single mutants performed at 16 °C, and 25 °C was determined. The fold difference in intragenic recombination frequency between 16 and 25 °C for wild type and each deletion was calculated to assess whether the reduction in gene conversion rate at cold temperatures is epistatic or synergistic with deleting *rqh1*, *exo1* or *sfr1* (Fig. 5). In wild-type crosses, gene conversion is 4.3-fold lower at 16 °C compared with 25 °C (*p* = 6.428 × 10^−12^, two-tailed Mann-Whitney U test). However, in *rqh1*Δ, *exo1*Δ and *sfr1*Δ crosses intragenic recombination levels are 7.2-fold (*p* = 1.402 × 10^−9^, two-tailed Mann-Whitney U test), 7.1-fold (*p* = 4.665 × 10^−11^, two-tailed Mann-Whitney U test) and 7.9-fold (*p* = 6.265 × 10^−7^, two-tailed Mann-Whitney U test) lower at 16 °C than at 25 °C, respectively (Fig. 5). The fold changes in overall CO frequency, and CO levels among Ade^+^ recombinants are largely unchanged or do not follow an obvious pattern (Table S4). Long-range strand resection and the action of strand exchange factors are potentially important for maintaining intragenic recombination frequency especially at colder temperatures, suggesting that these processes possibly are temperature-sensitive.

## Discussion

Generally, meiosis happens faster at higher temperatures, this has been observed in multiple (model) organisms (Bennett 1977; De La Peña et al. 1980; Stefani and Colonna 1996; Bomblies et al. 2015). However, this gain in developmental speed comes at the expense of fidelity indicated by cellular abnormalities and reduced reproductive success at higher temperatures (Loidl 1989; Stefani and Colonna 1996; Bomblies et al. 2015). In addition to pathological changes to the chromosome axis (Loidl 1989; Higgins et al. 2012; Morgan et al. 2017), the processing of recombination intermediates has been highlighted as a temperature-sensitive mechanism. The ability of meiotic RecA-family protein isolated from mouse and lily to support D-loop formation in vitro is decreased at higher temperatures (23 °C against 33 °C for lily, and 33 °C against 37 °C for mouse) (Hotta et al. 1985). This temperature preference of meiotic recombinase protein isolated from mouse aligns nicely with the temperature of mammalian testes in the scrotum, which tends to be ~ 5 °C lower than the normal body temperature of ~ 37 °C. Indeed, artificial cryptorchidism generated by surgically transplanting testes into the abdominal cavity of rats (i.e. raising testis temperature to body temperature in an in vivo system) causes substantial drops in recombination activity (Hotta et al. 1988).

The environmental temperature regime during sexual reproduction also influences meiotic recombination outcome (Bomblies et al. 2015). Temperature effects on recombination are largely species-specific and can manifest in different ways: (I) CO frequencies follow a U-shaped distribution in *Drosophila* or *Arabidopsis* (Plough 1917; Lloyd et al. 2018), where CO recombination is highest at the more extreme temperatures within the fertile range, (II) CO frequency increases with increasing temperature in *C. elegans* (Rose and Baillie 1979), (III) in grasshoppers CO frequency decreases with increasing temperature (Church and Wimber 1969) and (IV) in *S. cerevisiae*, *Hordeum vulgare* (barley) and *Secale cereale* (rye) overall CO frequency is maintained (De La Peña et al. 1980; Higgins et al. 2012; Zhang et al. 2017). In the latter situation, CO positioning can be altered, as demonstrated for budding yeast and barley (Higgins et al. 2012; Zhang et al. 2017). In *S. cerevisiae* this has largely been explained by DSB hotspot activation varying with temperature (Zhang et al. 2017).

In fission yeast, changes in environmental temperature during sexual reproduction affect the duration of mating, meiosis and sporulation (Fig. 1), as well as the meiotic recombination outcome tested at several versions of a genetic interval containing multiple different *ade6* heteroalleles (Fig. 3). The fertile range of *Sz. pombe* extends from ~ 10 to ~ 33 °C (Fig. 1). The duration of sexual reproduction lasts from 2 weeks at 11 °C to 48 h at 33 °C (Fig. 1). The CO frequency changes over temperature observed between *ura4*^+^-*aim2* and *his3*^+^-*aim* (Fig. 3d–f) do definitely not follow a U-shaped distribution like in *Drosophila* or *Arabidopsis* (Plough 1917; Lloyd et al. 2018), but are similar to *C. elegans* (Rose and Baillie 1979) where CO rates tend to be lower at low temperatures (Fig. 3d–f). Interestingly, gene conversion frequency at various different *ade6* heteroalleles shows strong changes with temperature within the fertile range, this is considerably more affected than COs and COs among intragenic events between *ura4*^+^-*aim2* and *his3*^+^-*aim* (Fig. 3). Within the fertile range, overall DSB levels appear to be similar at 16 °C and 33 °C in fission yeast as estimated by immunocytochemistry of Rec7-GFP and Rad11-GFP (Fig. 4). We counted an average of 14–18 foci/nucleus for both cytological markers in Hop1-positive nuclei; Hop1-staining of the meiotic chromosome axis was used to determine that the analysed nuclei were at the appropriate stage with the highest recombination events (Lorenz et al. 2004; Brown et al. 2018). This is similar to average focus counts for Rec7-GFP and Rad51 in a previous study (Lorenz et al. 2006), and is in line with an estimated ~ 60 DSB events/nucleus/meiosis (Fowler et al. 2014) considering that single-DSB events might cluster together (Fowler et al. 2018). Previously, temperature-induced changes in physical DSB formation at an *ade6* hotspot allele have been observed in a unisexual and artificially synchronized meiotic system using mutant *pat1* alleles, with DSB frequency being notably lower at 25 °C than at 34 °C (Hyppa et al. 2014). These discrepancies can have several technical sources, such as higher synchrony in meiotic cultures of *pat1* strains (Loidl and Lorenz 2009), and the unisexuality of the *pat1* synchronization tool affecting various meiotic processes (Bähler et al. 1991; Chikashige et al. 2004; Hyppa and Smith 2010; Hyppa et al. 2014) resulting in different readouts compared with normal zygotic or azygotic meiosis. Additionally, these discrepancies can point towards genuine biological differences, i.e. that DSB frequency is indeed altered between 25 and 34 °C at particular *M26*-type *ade6* hotspots, which might not be detectable in recombination outcome due to CO homeostasis and/or CO invariance (Hyppa and Smith 2010; Kan et al. 2011), whereas the more drastic reductions in gene conversion frequencies seen at 16 °C are caused by inefficiencies in DSB processing (Fig. 5). These two observations are also not mutually exclusive and could well overlap over the full fertile range. Further in-depth experimentation will be required to determine the exact contribution of these mechanisms to the thermotolerance of *Sz. pombe* meiosis. Considering that *M26*-type hotspots require the transcription factor Atf1-Pcr1 for full activation (Kon et al. 1997), any temperature-induced changes could also be caused by alterations in Atf1-Pcr1 recruitment to the hotspot sequence; this remains to be tested experimentally. In contrast to *S. cerevisiae* where ~ 80% of DSBs change location at different temperatures (14, 30 and 37 °C) (Zhang et al. 2017), only 17 out of 288 DSB hotspots behaved differentially between 25 and 34 °C in *Sz. pomb*e (Hyppa et al. 2014); it is, thus, less likely that activation of different hotspots is a major confounding factor in changing of recombination frequency at a given site in *Sz. pombe*. Considering that overall CO frequency between *ura4*^+^-*aim2* and *his3*^+^-*aim* is only moderately affected by temperature in a given interval, whereas gene conversion rates at *ade6* change massively, a switch from interhomologue to intersister recombination is also an unlikely factor, since it would affect intergenic COs and intragenic recombination to a similar extent. This discrepancy between gene conversion and CO frequency changes in relation to environmental temperature needs to be resolved. Our observation that strand resection, strand exchange and/or branch migration seem to be compromised at colder temperatures (Fig. 5), indicates that inefficiencies in these processes impair the conversion of point mutations in *ade6* alleles to wild-type *ade6*^+^. Nevertheless, mature recombination intermediates (D-loops, Holliday Junctions) can be formed between homologous chromosomes at these sites even at low temperatures, thus maintaining CO frequency. Furthermore, recombination monitored at the *ade6-M375* non-hotspot allele is less sensitive to temperature changes than that involving an *M26*-type *ade6* hotspot (Fig. 3, Table S4). This indifference to temperature could be a manifestation of CO invariance, which has been suggested as an explanation for a stronger drive towards interhomologue recombination in regions of low meiotic recombination frequency (Hyppa and Smith 2010).

One of the most obvious traits of fission yeast meiosis is the lack of a synaptonemal complex (Loidl 2006). Because extreme temperatures affect synaptonemal complex structure (Loidl 1989; Higgins et al. 2012; Bilgir et al. 2013), and mutants of components of the synaptonemal complex or the meiotic chromosome axis show differential phenotypes at different temperatures (Börner et al. 2004; Penedos et al. 2015), it has been suggested that the synaptonemal complex is a salient factor in determining the meiotic thermotolerance of a species (Morgan et al. 2017). In *Sz. pombe*, though, meiotic thermotolerance seems to rely on biochemical activities supporting DSB formation (Hyppa et al. 2014), recombination intermediate formation (DNA strand exchange) and/or stabilization (Fig. 5). Although, *Sz. pombe* lacks a fully-fledged synaptonemal complex, components of its linear elements are homologous to lateral element proteins of the synaptonemal complex (Lorenz et al. 2004; Loidl 2006). It is rather difficult to test whether aspects of meiotic thermotolerance are conferred by the linear elements, because deletion of linear element genes strongly reduces meiotic recombination or, in most cases, abolishes it completely (Ellermeier and Smith 2005; Davis et al. 2008; Latypov et al. 2010; Estreicher et al. 2012). However, *rec10–144*, a hypomorphic mutant of the main linear element component *rec10*, shows some temperature sensitivity in intragenic recombination (Pryce et al. 2005). This suggests that in addition to DSB formation and processing, linear elements might also play a role in meiotic thermotolerance.

Despite more than 100 years of research (Plough 1917), we still only have a basic understanding of how environmental parameters influence meiotic recombination outcomes in different species. Environmental temperature grossly affects the speed of sexual reproduction (Bennett 1977) and recombination levels in several species (Plough 1917; Rose and Baillie 1979; Bomblies et al. 2015; Lloyd et al. 2018), likely by changing the frequency of DSB formation at a given site (Hyppa et al. 2014), the positioning of the initial DSB (Higgins et al. 2012; Zhang et al. 2017) and/or dynamics of DSB repair (Modliszewski et al. 2018; this study). Here, we define the fertile range over temperature in the fission yeast *Schizosaccharomyces pombe*, and determine that this stipulates the pace of sexual reproduction and the level of recombination, gene conversion events in particular. At least in part, the latter seems to be driven by temperature-sensitive steps of recombination intermediate processing downstream of DSB formation, such as DNA strand exchange and/or branch migration. Our study highlights the importance of the interplay between intrinsic and environmental factors in shaping the genetic diversity of a given population, and the need for further experimentation to elucidate the cellular mechanisms underpinning meiotic thermotolerance.

## Electronic supplementary material

ESM 1 (XLSX 39 kb)

## Abbreviations

aim: artificially introduced marker
DSB(s): DNA double-strand break(s)
DNA: deoxyribonucleic acid
CO(s): crossover(s)
GC-CO: gene conversion associated with a crossover
GC-NCO: gene conversion associated with a non-crossover
GFP: green fluorescent protein
ME: malt extract
NCO(s): non-crossover(s)
YE: yeast extract
YNG: yeast nitrogen base glutamate
